# Supplementary material for: The Geographic Context of Racial Disparities in Aggressive Endometrial Cancer Subtypes: Integrating Social and Environmental Aspects to Discern Biological Outcomes
Source: Int J Environ Res Public Health. 2022 Jul 15;19(14):8613. doi: 10.3390/ijerph19148613 (PMC9320863; doi:10.3390/ijerph19148613)
Supplement: Supplementary file 1 [file ijerph-19-08613-s001.zip › ijerph-1779953-supplementary.pdf]

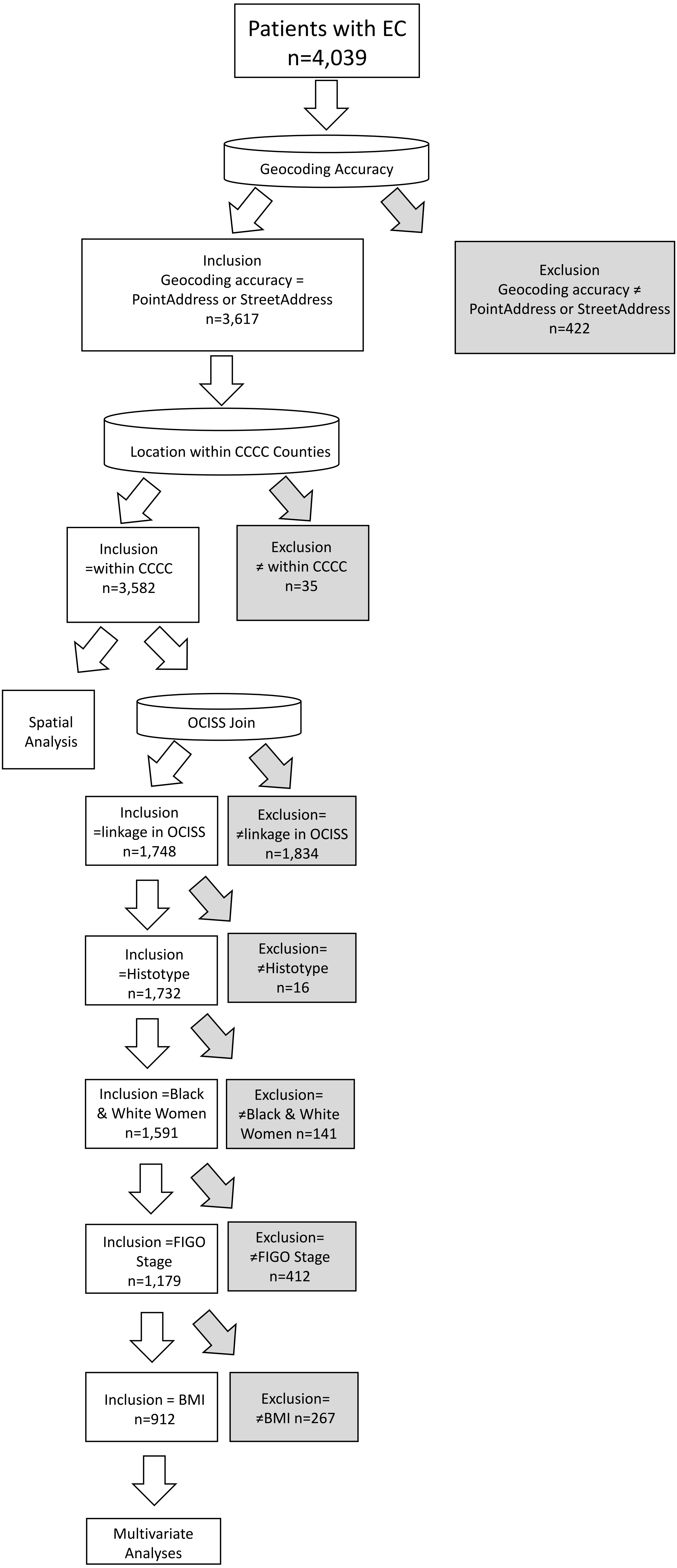
Patients with EC
n=4,039
Geocoding Accuracy
Inclusion
Geocoding accuracy =
PointAddress or StreetAddress
n=3,617
Exclusion
Geocoding accuracy ≠
PointAddress or StreetAddress
n=422
Location within CCCC Counties
Inclusion
=within CCCC
n=3,582
Exclusion
≠ within CCCC
n=35
Spatial Analysis
OCISS Join
Inclusion
=linkage in OCISS
n=1,748
Exclusion=
≠linkage in OCISS
n=1,834
Inclusion
=Histotype
n=1,732
Exclusion=
≠Histotype
n=16
Inclusion =Black & White Women
n=1,591
Exclusion=
≠Black & White Women n=141
Inclusion =FIGO Stage
n=1,179
Exclusion=
≠FIGO Stage
n=412
Inclusion = BMI
n=912
Exclusion=
≠BMI n=267
Multivariate Analyses

Figure S1: Inclusion and Exclusion Flowchart

Of the 4,039 women in our dataset, 442 were excluded due to the inability to geocode their location. Another 35 women were excluded as they resided outside of the CCCC catchment area. The resulting 3,582 individuals were used in spatial analyses. For multivariate regression and survival analyses, 1,834 women were removed for missing direct linkage with the OCISS dataset, 16 women removed for missing a histotype, 141 were removed for not self-reporting races as Black or White, 412 were removed for missing a disease stage, and 267 were removed for missing a BMI characteristic, height and/or weight.

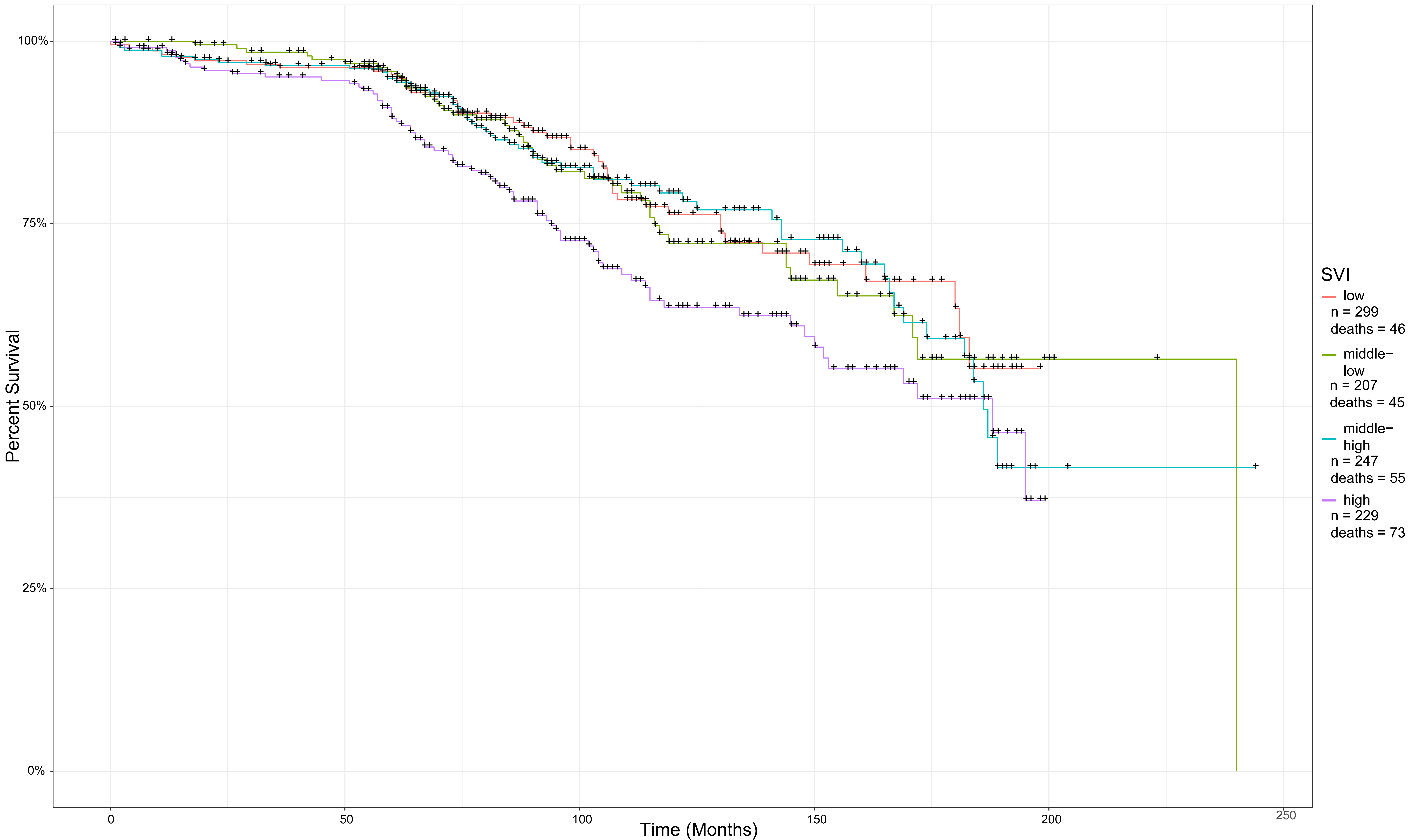
100%
75%
50%
25%
0%
Percent Survival
0
50
100
150
200
250
Time (Months)
SVI
low
n = 299
deaths = 46
middle−
low
n = 207
deaths = 45
middle−
high
n = 247
deaths = 55
high
n = 229
deaths = 73

Figure S2: Association of SVI and Survival

SVI, as a categorical variable of quartiles low, middle-low, middle-high, and high, is associated with poorer survival. The percent of survival over time in months is described for all four quartiles. Compared to the lowest SVI quartile, the highest SVI has significantly poorer survival over time

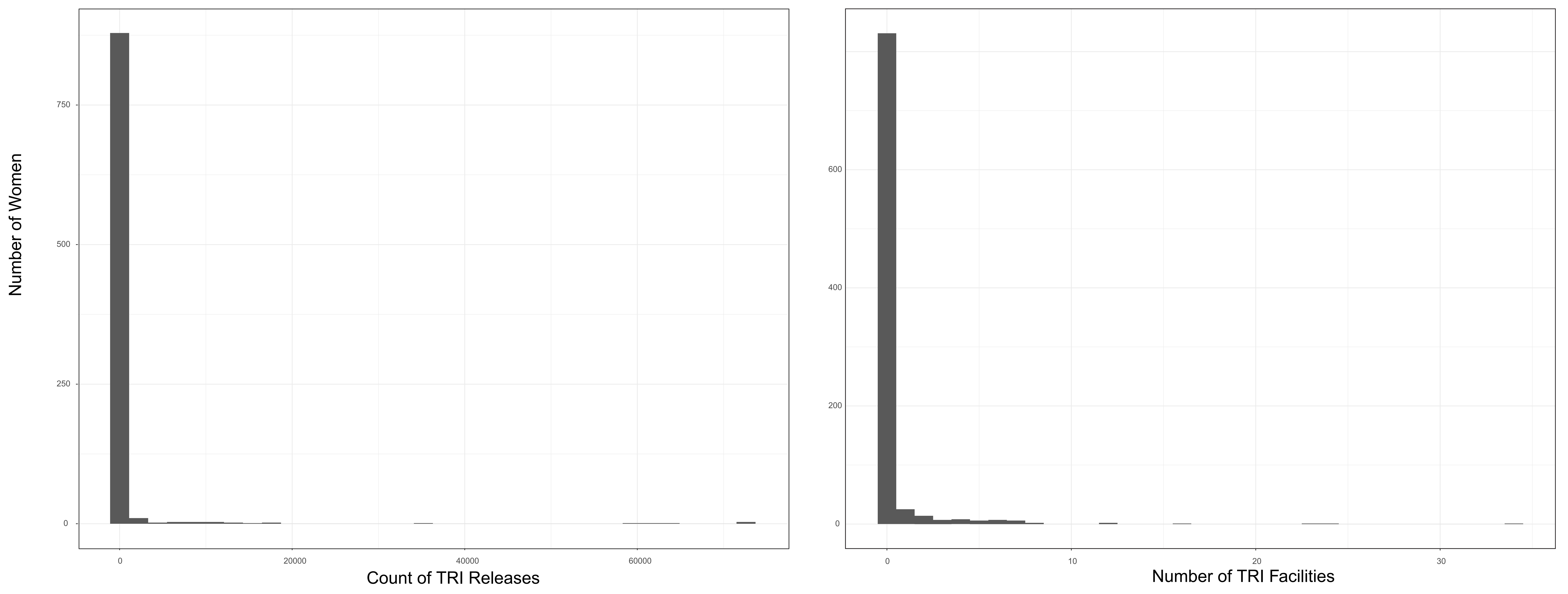

Number of Women
750
500
250
0
0
20000
40000
60000
Count of TRI Releases
600
400
200
0
0
10
20
30
Number of TRI Facilities

Figure S3: Distribution of TRI Count and TRI Density

Panel A notates the distribution of the count of TRI releases and Panel B is the number of TRI facilities for each woman studied. Most of the women studied did not live near a TRI facility and therefore, had little to no exposure.

Supplemental Table S1. Multivariable Models that Associate with Histotype with Continuous Variables

	Full Model SVI			Full Model TRI Count			Full Model TRI Densiy	
	**OR (95% CI)**	**P-value**		**OR (95% CI)**	**P-value**		**OR (95% CI)**	**P-value**
**SVI**	2.14 (1.26, 3.63)	0.0047*	**TRI Count**	1.06 (0.99, 1.15)	0.08	**TRI Density**	1.00 (1.00, 1.00)	0.3

*P-value < 0.05

Supplemental Table S2. Overall Deaths Multivariable Analysis with Continuous Variables

	Full Model SVI			Full Model TRI Count			Full Model TRI Density	
	**Hazard Ratio (95% CI)**	**P-value**		**Hazard Ratio (95% CI)**	**P-value**		**Hazard Ratio (95% CI)**	**P-value**
**SVI**	1.86 (1.17,2.94)	8.33E-03*	**TRI Count**	0.95 (0.87,1.04)	0.25	**TRI Density**	1.00 (1.00, 1.00)	0.48

*P-value < 0.05

Supplemental Table S3: Race Stratified Survival Model of Univariate SVI

	Black Women		White Women	
	**OR (95% CI)**	**P-Value**	**OR (95% CI)**	**P-Value**
Continuous				
SVI	2.33 (0.73, 7.70)	0.16	1.99 (1.13, 3.50)	0.018
Categorical				
SVI High	1.34 (0.52, 3.48)	0.54	1.86 (1.18, 2.96)	0.0077*
SVI Medium-High	1.55 (0.63, 3.88)	0.35	0.87 (0.53, 1.42)	0.58
SVI Medium-Low	1.34 (0.52, 3.48)	0.92	1.34 (0.83, 2.18)	0.23

*P-value < 0.05

Appendix for Abbreviations

BMI: Body Mass Index
CCCC: Case Comprehensive Cancer Center
CDC: Centers for Disease Control and Prevention (U.S.)
CSR: Complete Spatial Randomness
EC: Endometrial Carcinoma
EMR: Electronic Medical Record
EPA: Environmental Protection Agency (U.S.)
FIGO: International Federation of Gynecology and Obstetrics
GIS: Geographic Information System
LMI: Local Moran's I
OCISS: Ohio Cancer Incidence Surveillance System
SVI: Social Vulnerability Index
TRI: Toxic Release Inventory
UGCoP: Uncertain Geographic Context Problem
UH: University Hospitals
